# CLARIFY registry launched in South Africa

**Published:** 2010-06

## Introduction

The CLARIFY registry (ProspeCtive observational LongitudinAl RegIstry oF patients with stable coronary arterY disease) is the first and largest international registry designed to increase knowledge and understanding of stable coronary artery disease (CAD).

Coronary artery disease remains the leading cause of death worldwide, therefore improving the understanding of the management and outcomes of these patients with stable CAD is paramount for reducing the CAD disease burden. Data currently available on presentation and management of patients with stable CAD is mainly from clinical trials or registries that often have stringent exclusion criteria, and therefore do not adequately represent populations with stable CAD seen in clinical practice (in terms of age, co-morbidity and treatment).

‘Despite the growing importance of heart rate in the treatment of CAD, there is little existing data on resting heart rate in patients seen in day-to-day clinical practice, so a registry of CAD patients involving the heart is long overdue, particularly as heart rate needs to be carefully measured’, commented study lead Prof Philippe Gabriel Steg, Bischat-Claude Bernard Hospital, Paris, France.

CLARIFY is therefore designed to characterise the demographic and clinical profile of patients with stable CAD and will reflect the entire spectrum of outpatients with stable CAD seen by cardiologists and primary-care physicians in daily clinical practice. The main aim will be to determine long-term prognostic factors in these patients, including the role of resting heart rate, with a view to developing a risk-prediction model, since heart rate is not yet a routine component of cardiovascular risk assessment, or in deciding whether treatment is indicated.

CLARIFY will involve a minimum of 30 000 outpatients with stable CAD from 40 countries worldwide, who will be followed for five years. South Africa is also participating in this international registry.

Prof DP Naidoo, University of KwaZulu Natal, the national coordinator for CLARIFY commented: ‘I am keen to see what this snapshot of the current care of patients with CAD in South Africa will show’. Patients are being recruited from both the private and the public sector in South Africa, from both metropolitan areas and outlying clinics, so the sample will be truly representative of current practice in South Africa.

Patient recruitment started around the world in October 2009. Commenting on behalf of Servier, who provided an educational grant for the registry, Pat Magagula is confident that South Africa will exceed expectations by recruiting over 500 patients.

